# Acupuncture treatment of acute urinary retention caused by varicella-zoster virus through by combining the mechanism of sacral neuromodulation: A rare case report

**DOI:** 10.1097/MD.0000000000036007

**Published:** 2023-12-01

**Authors:** Zilong Tan, Jianwu Shen

**Affiliations:** a Department of Urology, Xiyuan Hospital, China Academy of Chinese Medical Sciences, Beijing, China.

**Keywords:** acupuncture treatment, acute urinary retention, case report, neurogenic bladder, varicella-zoster virus

## Abstract

**Rationale:**

Urinary dysfunction triggered by varicella-zoster virus (VZV) attacking the nervous system seriously affects the quality of life of patients and may even cause irreversible damage to the urinary system. This a 62-year-old man with acute urinary retention triggered by VZV, who was cured after acupuncture treatment. The rational application of acupuncture therapy to promote the recovery of bladder contraction function can effectively relieve the symptoms of dysuria, shorten the course of the disease.

**Patient concerns:**

Symptoms included dysuria and distension of the bladder area secondary to postherpetic herpes zoster, with significant pressure and pain in his lower abdomen, accompanied by cutaneous herpes distributed over the sacral region.

**Diagnoses:**

The case was diagnosed as acute urinary retention (Neurogenic Bladder). Ancillary tests include urodynamic examination, Doppler ultrasound, urodynamic tests are the preferred diagnostic method and suggest: no contraction of the detrusor muscle is seen during voiding, and voiding occurs in an The absence of abdominal pressure-assisted micturition and repeated attempts to pass urine suggests detrusor weakness; residual urine suggests a severe bladder emptying disorder. Doppler ultrasound suggested overfilling of the bladder, and 1153 mL of residual urine was seen in the bladder after voiding.

**Interventions:**

The patient developed sacral herpes and dysuria and was treated with oral antiviral drugs on the 12th day of illness. But his urinary difficulty did not improve but gradually worsened, resulting in acute urinary retention, and he then turned to the acupuncture treatment, innovative approach combined the mechanism of action of sacral neuromodulation with traditional Chinese medicine theory.

**Outcomes:**

The duration of acupuncture treatment totaled 12 weeks; he was able to urinate on her own and her symptoms completely disappeared. No other adverse and unintended events occurred during treatment.

**Lessons:**

This study demonstrates that acupuncture is safe and effective in the treatment of acute urinary retention caused by VZV, which is worth recommending as a conservative treatment. Moreover, we found that the early intervention and full-term treatment with acupuncture is particularly important, provided that the right key acupoints are selected.

## 1. Introduction

Herpes zoster (HZ) is a neurologic skin infection caused by reactivation of varicella-zoster virus (VZV) latent in the human body. Infected individuals are susceptible to pathological changes such as sensory root ganglion, meningeal or spinal cord inflammatory injury,^[1–3]^ and their presentation is characterized by a unilateral, painful blister-like rash in a restricted dermatomal distribution. Recent epidemiological surveys have shown that HZ occurs in an average of 4.76 million people per year in China,^[4,5]^ with some patients suffering from disorders of the urinary tract and gastrointestinal tract due to the invasion of sympathetic and parasympathetic fibers by VZV from the posterior root ganglia of the spinal cord. Involvement of internal organs by the virus is less common, and the clinic usually starts with cutaneous symptoms, among which Acute Urinary Retention (AUR) due to limitation of urinary function involving the bladder is also known as Elsberg’s syndrome,^[6]^ which belongs to one of the types of neurogenic bladder. Voiding dysfunction caused by herpes zoster was first described by Davidsah in 1890, and since then more than 200 cases have been reported in the literature, and more recently, 423 cases of HZ in a large institution were also reviewed, with herpes zoster infections in the sacral region accounting for 8% of all herpes zoster infections, and it was found that 4.02% of these voiding dysfunctions were associated with VZV infection.^[7]^ Because of the low number of reported cases of ES, clinicians are not very cognizant of it and are unable to perform timely imaging or pathogenetic tests for a definitive diagnosis, so outpatient clinics often misdiagnose AUR patients with dyspareunia due to causes such as urethral obstruction because of ignoring their history of herpes zoster. Also because VZV visceral disseminated infection can also occur in immunocompetent people,^[8,9]^ and even patients do not always show skin symptoms after infection,^[10]^ the onset of which is more insidious. All of the above reasons largely increase the diagnostic difficulty, which makes it difficult for clinicians to treat HZ as one of the triggering causes of acute urinary retention. ES is usually treated as an idiopathic inflammatory condition,^[11]^ and this misdiagnosed or poorly treated condition can allow the resulting detrusor muscle weakness to persist for 8 weeks or longer, with risks of leading to bladder rupture and postrenal disease.^[12–14]^ Nowadays, the management of detrusor muscle weakness type of urinary retention is a challenge in the clinic, and its main treatments include non-conservative surgical treatments and pharmacological conservative treatments; however, due to the limitations of these therapies, acupuncture as an effective and complementary therapy is a therapeutic modality worthy of being promoted and practiced globally. In this case study, we investigate the effect of acupuncture treatment on the recovery of bladder detrusor contractility after VZV-induced urinary dysfunction, based mainly on the evaluation of the patient’s urodynamic changes.

## 2. Case report

### 2.1. A 62-year-old man, presented on July 27, 2022

**Complaint:** Difficulty in urination for 12 days with progressive aggravation for a week.

**History:** 12 days ago, he had erythema, blisters and localized ulcers on the skin and mucous membranes of the left caudal sacral region without any obvious triggers, accompanied by pain and occasional difficulty in urination, so he consulted the dermatology clinic and was diagnosed with herpes zoster, and was orally administered with famciclovir tablets, 75 mg/d, and pregabalin capsule, 300 mg/d. After 4 days of medication, the skin lesions and pain symptoms almost disappeared, and on the 5th day, the weakness of urination, waiting to urinate, and dysuria were aggravated, accompanied by abdominal distension, stopping of anal defecation, occasional nausea and vomiting, and no fever and chills and other discomforts, so she came to seek acupuncture treatment. Current Symptoms: floating face, fatigue and tiredness, shortness of breath and chest tightness, low voice, dullness and low food intake, distension and discomfort in the upper pubic symphysis region, difficulty in urination and constipation.

**Past history:** No previous history of benign prostatic hyperplasia, urinary sensation, volume and frequency were normal before the onset of the disease.

**Personal and Family history:** Family history of hereditary disease was denied.

**Physical examination:** On admission, vital signs were T: 36.4°C; R: 18 beats/min; HR: 65 beats/min; BP: 118/75 mm Hg. The patient had a painful face with a distended lower abdomen, no gastrointestinal peristaltic waves or abdominal wall varicose veins were seen. Positive lower abdominal tenderness, palpable distended bladder, diminished bowel sounds, and decreased sensation in the perianal region. Rectal palpation: no enlargement or abnormal nodules in the prostate. Dermatologic examination: the patient’s left sacral region showed clusters of blisters crusted in patches of varying sizes, and the lesions had recovered roughly and did not exceed the median line; tenderness (+/-).

**Figure 1.**
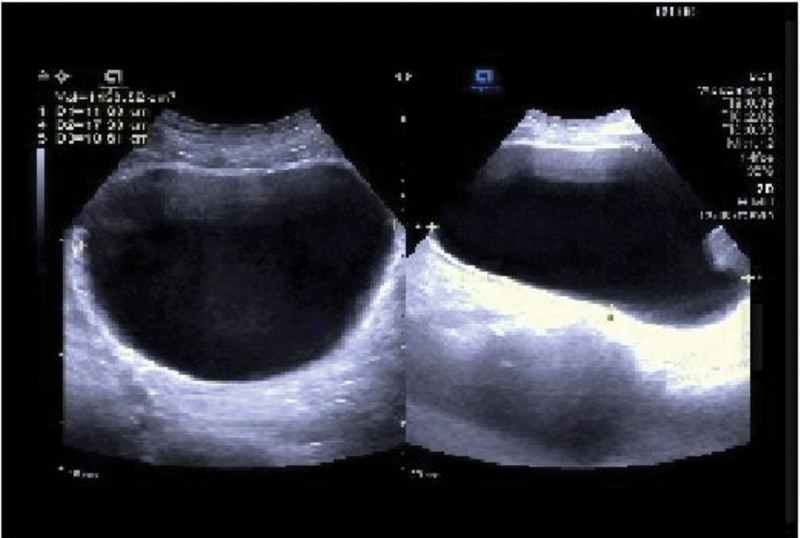
Urologic Doppler ultrasound showed: normal prostate size (3.9 cm*2.9 cm*2.6 cm); post voiding bladder residual urine: 1153 mL.

**Figure 2.**
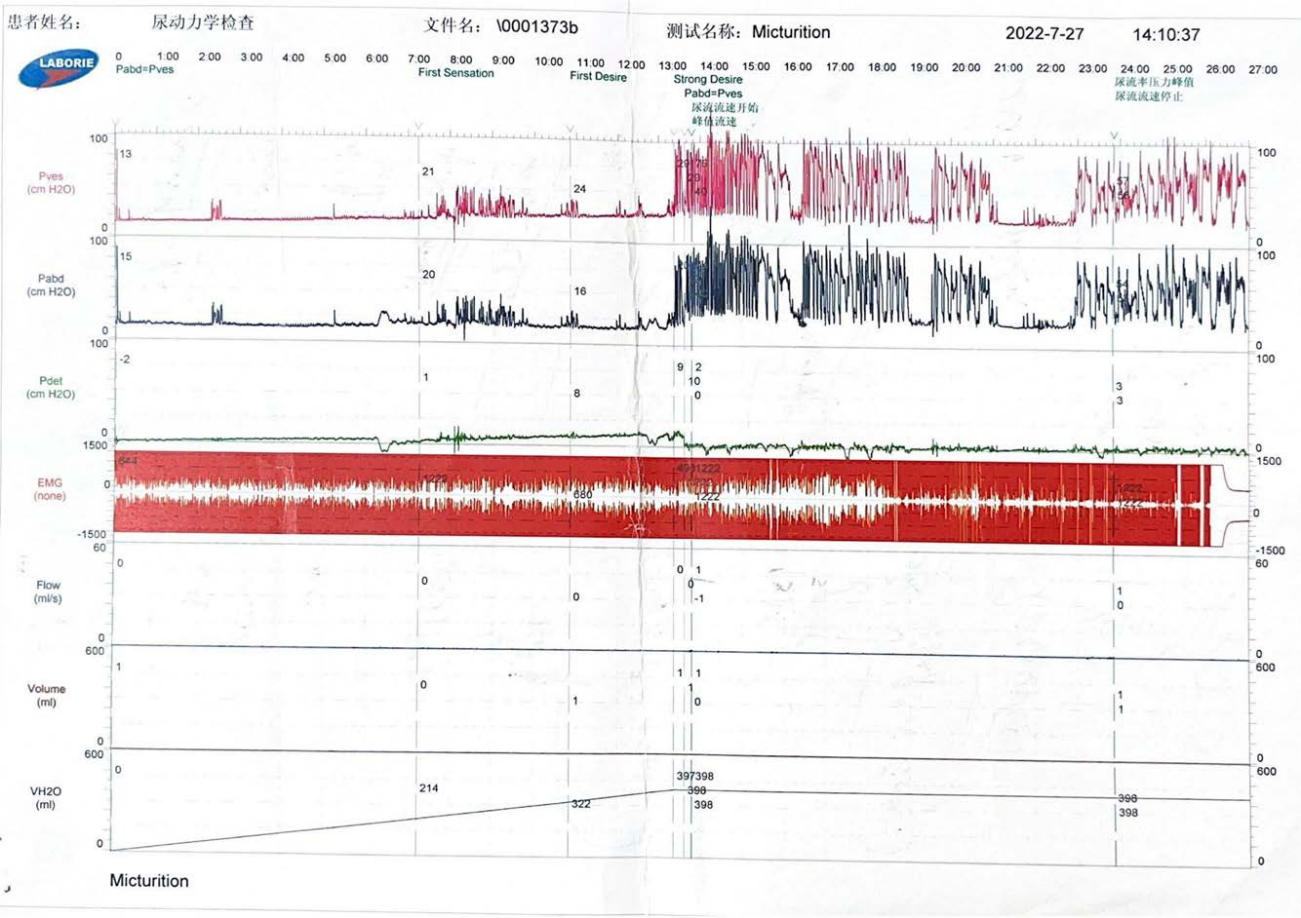
(i) In the early stage of bladder perfusion in the storage period, the intravesical pressure was 15.0 cmH_2_O, the initial sensory urinary volume was 255 mL, the intravesical pressure was 21.5 cmH_2_O, and the normal urinary volume was 315 mL, and the intravesical pressure was 26 cmH_2_O, which suggests that bladder compliance is normal, and the bladder urinary sensation is normal. (ii) Functional capacity of the bladder was greater than 400 mL, suggesting normal cystometric volume; (iii) No contraction of the detrusor muscle was seen during the voiding period, and voiding was in the mode of abdominal pressure-assisted voiding, and no urine was discharged in the repeated attempts, suggesting detrusor muscle incompetence; (iv) Residual urine was greater than 400 mL, suggesting a severe obstruction of bladder emptying.

**Complementary checkup:** The skin lesion swabs were obtained by the patient during a pre-admission visit to the dermatology department for polymerase chain reaction testing (PCR) for VZV: positive for VZV. Laboratory tests including biochemical analysis, complete blood count, serum immunoglobulins (IgG, IgA, and IgM), infectious disease screen, and stool examination were normal. Serum total prostate-specific antigen (TPSA): 4.07 µg/L. Urinalysis and urine bacterial culture (-); urologic Doppler ultrasound showed: normal prostate size (3.9 cm*2.9 cm*2.6 cm); postvoiding bladder residual urine: 1153 mL (Fig. 1); urodynamic examination results on July 27, 2022 suggested that: no contraction of detrusor muscle was seen during the voiding period, voiding was in the mode of abdominal pressure-assisted voiding, and there was no urinary discharge in repeated attempts. suggestive of weakness of bladder detrusor contraction; residual urine suggestive of severe impairment of bladder emptying (Fig. 2). Magnetic resonance imaging (MRI) of the brain, lumbar and sacral spine, electrocardiogram, chest CT, and Doppler ultrasound of the epigastric region showed no significant abnormalities.

Treatment: The final diagnosis of acute urinary retention (Neurogenic Bladder) was made by combining his history and examination findings. On day 1 of admission, he underwent urethral intubation. On day 5 of admission, an attempt to remove the catheter ended in failure. Subsequently, he was started on electroacupuncture (EA), acupoints chosen were bilaterally BL33, BL32, CV3, CV2, SP6. Localization: Refer to the National Standard of the People’s Republic of China (GB/T12346-2021) “Names and Localization of Acupoints,” in which the “Sacral Neuromodulation Localization Method” should be used for BL33 (along the midline of the patient’s sacrum, measuring 9 cm upward from the tip of the coccyx and 2 cm aside, the posterior sacral foramen of S3 corresponds to the surface of the body) or the “Sciatic Notch Positioning Method” (the horizontal line of the S3 sacral foramen was made at the intersection of the lines of the situs notch and the midline of the sacrum on both sides, and the intersection was opened by 2 cm on both sides.). The needles were 0.30 mm*75 mm (3 inches), 0.40 mm*100 mm (long needle) Zhongyan Taihe Brand Disposable Acupuncture Needles (Beijing Zhongyan Taihe Medical Equipment Co., Ltd., Beijing, China); and the Electroacupuncture instrument was the Indy Brand KWD-808I Electroacupuncture Therapeutic Instrument (Changzhou Yingti Electronic Medical Equipment Co., Ltd., Changzhou, China). The BL33, BL32, and SP6 used 3-inch filliform needle; BL33 was positioned in the above way, and the needle was inserted 1 cm outside and above the body surface of the 3rd post-sacral foramen, and stabbed obliquely in an inward and downward direction at an angle of approximately 45° to 60° to the patient’s skin for 45 to 55 mm, and the other acupoints were stabbed directly with acupuncture needles for 30 to 40 mm; the CV3 and CV2 were pierced vertically for 70mm using a long needle. All acupoints were evenly lifted and inserted and twisted so that the patients felt arrival of Qi, connected EA instrument electrodes on the needle handles of BL33 and BL32 bilaterally, adjusted the position of sacral hole acupuncture needles, and mainly asked the patients whether there was a sacral nerve response [rectal tugging sensation, radiating sensation to the perineum, opening and closing movement of the anus area (bellows-like sensation), and metatarsal curvature of the ipsilateral big toe or other toes], and then electrified the patient for 30 minutes after a response was made. If the nerve response is not obvious after inserting the first acupuncture needle, insert another acupuncture needle at its edge, electrify the second acupuncture needle, and if the nerve response can be detected, remove the first acupuncture needle, treat with the second acupuncture needle, and repeat the above procedure until a suitable response location is found. EA parameters: sparse-dense wave, frequency 50 Hz, initially applied current strength 1 to 5 mA, gradually increasing the current strength to the extent tolerated by the patient. Needle retention time and treatment course: 30 minutes per needle retention, EA treatment every other day, 3 times per week, a treatment cycle of 4 weeks, the total number of treatments is 12. EA is performed by a certified acupuncturist with fifteen years of experience.

**Figure 3.**
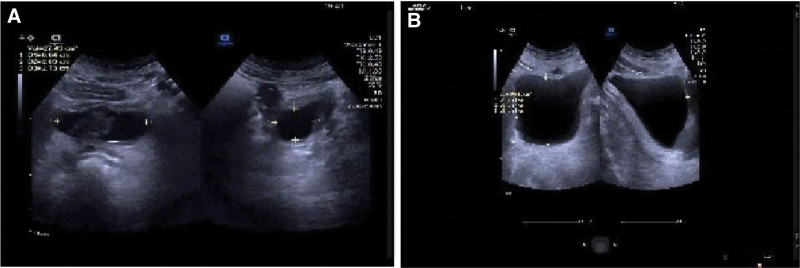
A. On September 22, 2022, a follow-up ultrasound of bladder residual urine showed 27 mL of residual urine in the bladder after urination. B. Urinary retention developed again after catheter removal, and the performed ultrasound showed a residual urine volume of 166 mL.

**Figure 4.**
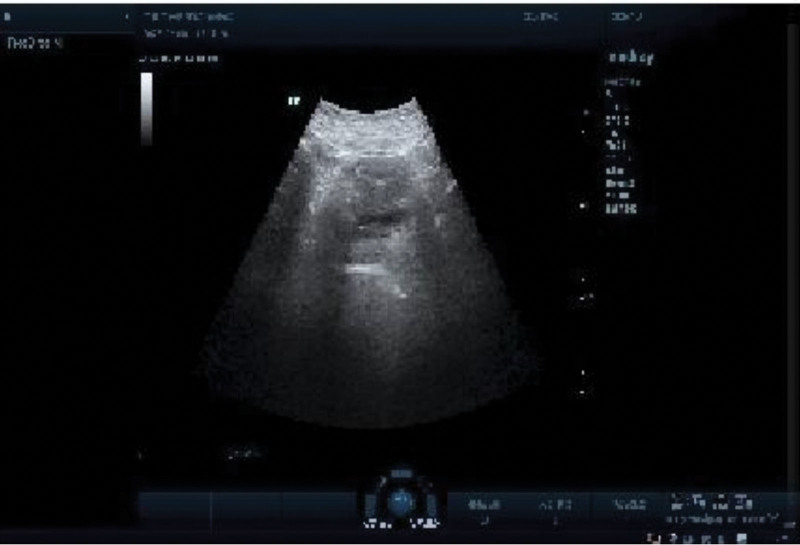
At the end of the subsequent treatment, he had fully recovered and no more residual urine appeared after voiding.

**Figure 5.**
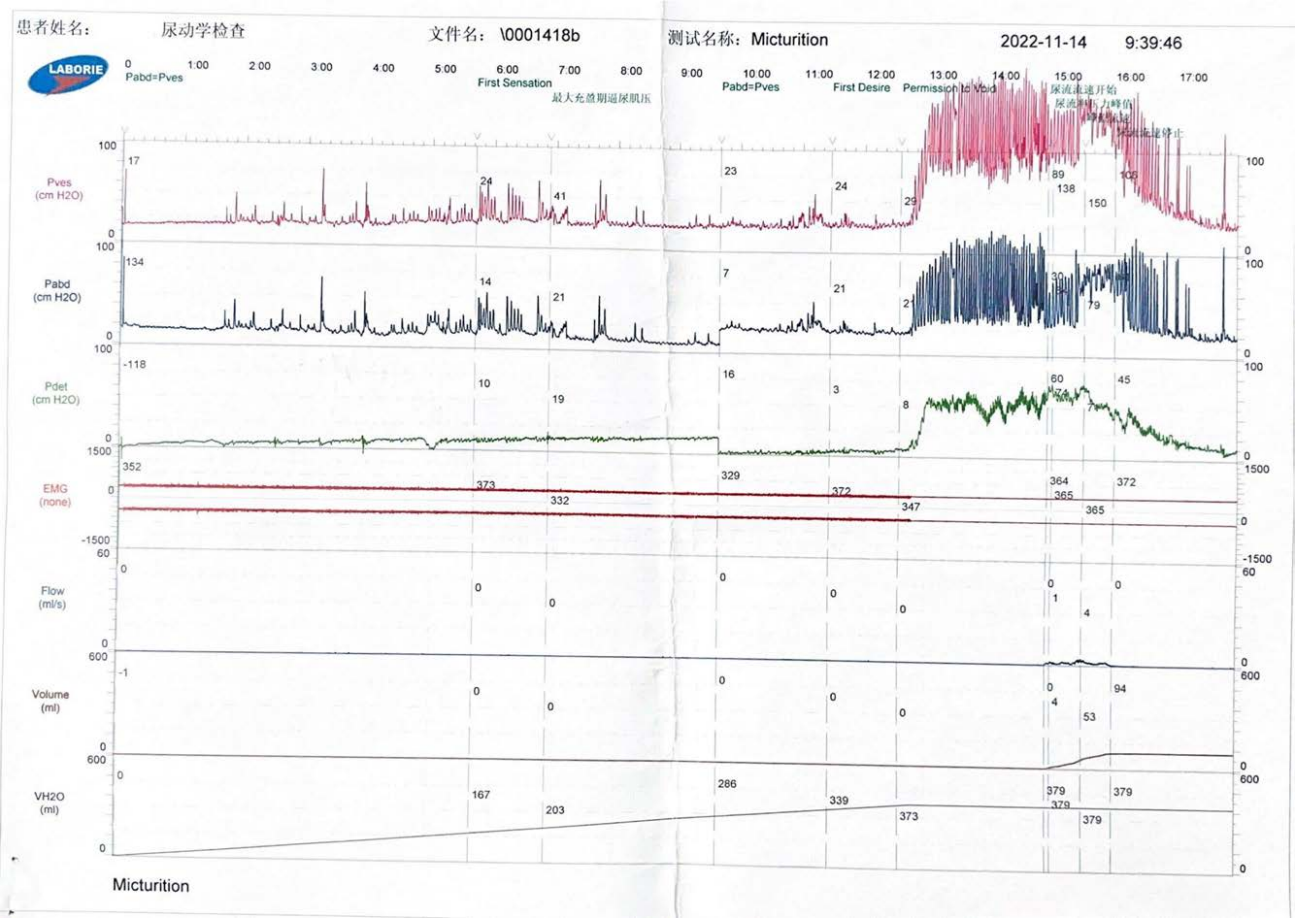
reexamination of urodynamics on November 14, 2022: (i) The intravesical pressure at the beginning of bladder perfusion in the storage phase was 18.5 cmH_2_O, the initial voiding volume was 165 mL, the normal voiding volume was 341 mL, and the intravesical pressure was 25.5 cmH_2_O, which suggested a normal urinary sensation of the bladder and a normal bladder compliance; (ii) The bladder functional volume was about 400 mL, suggesting normal cystometric volume; (iii) From the beginning to the end of perfusion, no involuntary contraction of the detrusor muscle was seen, and no urge incontinence was seen; (iv) Detrusor contraction was seen during the voiding period, and voiding was in the mode of abdominal pressure assisted voiding with a maximal detrusor contraction force of 71.5cmH_2_O, and the maximal urinary flow rate was 4 mL/s, and voiding was in the state of high pressure and low flow, suggesting that there might be an obstruction in the outflow tract of the bladder; (v) The residual urine was about 42 mL, suggesting that the bladder emptying was fair.

Results and Follow-Up: During the first 3 weeks of EA treatment, he developed a transient urinary tract infection, probably due to his prolonged urinary retention, which resolved spontaneously without antimicrobial treatment. After 9 weeks of treatment, the patient feels strong urination, gas and bowel movement are more fluent than before the treatment, and the skin sensation around the perineum is gradually restored. On September 22, 2022, a follow-up ultrasound of bladder residual urine showed 27 mL of residual urine in the bladder after urination (Fig. 3A). Due to the strong request to remove the bladder catheter, however, urinary retention developed again after catheter removal, and the performed ultrasound showed a residual urine volume of 166 mL (Fig. 3B). Therefore, he received the same electroacupuncture treatment again on an outpatient basis for 3 weeks. At the end of the subsequent treatment, he had fully recovered and no more residual urine appeared after voiding (Fig. 4). Immediately following the urodynamic examination, it was suggested that (reexamination of urodynamics on November 14, 2022): (i) the intravesical pressure at the beginning of bladder perfusion in the storage phase was 18.5 cmH_2_O, the initial voiding volume was 165 mL, the normal voiding volume was 341 mL, and the intravesical pressure was 25.5 cmH_2_O, which suggested a normal urinary sensation of the bladder and a normal bladder compliance; (ii) the bladder functional volume was about 400 mL, suggesting normal cystometric volume; (iii) from the beginning to the end of perfusion, no involuntary contraction of the detrusor muscle was seen, and no urge incontinence was seen; (iv) detrusor contraction was seen during the voiding period, and voiding was in the mode of abdominal pressure assisted voiding with a maximal detrusor contraction force of 71.5 cmH_2_O, and the maximal urinary flow rate was 4 mL/s, and voiding was in the state of high pressure and low flow, suggesting that there might be an obstruction in the outflow tract of the bladder; (v) the residual urine was about 42 mL, suggesting that the bladder emptying was fair (Fig. 5). At the later follow-up of 6 and 12 months, all symptoms had completely disappeared and there was no longer any significant bladder residual urine on regular follow-up ultrasound, which the patient was very satisfied with. No adverse reactions or unintended events related to the described treatments were observed during the treatment period.

## 3. Discussion

VZV is a neurophilic α-herpesvirus^[15]^ that follows T cells to spread to the skin and establish an incubation period in ganglia,^[16]^ and when activated, it invades the micturition reflex nerves and thus causes urinary retention in the following ways^[17]^: VZV invades the sacral dorsal root ganglia of the spinal cord, the nerve roots, and the peripheral nerves undergoing neuroinflammatory changes, which cause disruption of the voiding reflexes; VZV directly invades bladder inner wall nerves leading to inflammatory lesions of the bladder, which ultimately triggers low contractility of the detrusor muscle; the perineal nerves and pelvic plexus nerves are damaged due to the destruction of VZV, which breaks the coordination of the functions of the detrusor muscle, the internal bladder sphincter, and the urethral sphincter, and affects the urinary excretion. In conclusion, ES is an infectious syndrome manifested by various signs of acute lumbosacral nerve root myelitis (e.g., urinary retention and lumbosacral sensory symptoms), which, in specific cases, results in inflammatory lesions of the nerve fibers involved in the voiding reflexes,^[18]^ and the viral etiology of the disease has been established.^[19]^ However, acute urinary retention occurring in adult males should also be differentiated or observed in combination with conditions such as benign prostatic hyperplasia, peripheral nerve diseases involving the sacral medulla (e.g., diabetic neurogenic bladder), and bacterial infections of the urinary system. When unexplained voiding dysfunction is observed, cutaneous herpes zoster around the genitals should be considered as the main point of differentiation.^[20]^

We dealt with a patient who presented with urinary retention and cutaneous herpes in the sacral region, and based on the symptoms, history of herpes zoster infection, ancillary investigations, and other etiologies that were ruled out, the patient fulfilled the clinically available diagnostic criteria, which ultimately established the conclusion of acute urinary retention (ES) due to VZV infection. Such patients are usually left with some degree of permanent neurologic impairment, very few recover fully, and the majority of those who do recover suffer from sequelae.^[21]^ There is no consensus on the treatment of ES, treatment for viral infections is limited, and corticosteroids have become one of the commonly prescribed medications.^[22]^ Intermittent catheterization is a therapeutic modality for temporary relief of acute illness, antiviral medication may be effective,^[23]^ and hormonal medication may be counterproductive by further accelerating the progression of HZ to visceral dysfunction. Several studies have reported that patients with ES may similarly develop chronic neurologic damage due to inappropriate treatment,^[24]^ so catheterization and conventional medication for ES are insufficient, and there is a need for improved therapeutic measures.

Acupuncture is an important part of Chinese medicine and is widely used as a non-pharmacological treatment to promote recovery from neurological dysfunction and pain relief. High-quality studies have demonstrated that acupuncture is effective in improving voiding disorders,^[25–27]^ and previous studies have confirmed the positive effects of acupuncture and electrical stimulation on bladder dysfunction after neurological injury.^[28–32]^ Based on the current understanding of the possible mechanisms of action of acupuncture to improve urinary condition, it can be considered to be applied to the regulation of neurological function in patients with herpes zoster urinary retention, and we chose to work on these points in our patients for the following reasons:

Firstly, the acupoints belong to the bladder meridian, the Ren meridian, and the spleen meridian. Chinese medicine believes that where the meridians pass through, the main treatment of the disease that they reach, the meridians are the place where the Qi and blood of the internal organs run, and needling the local tissues can regulate the qi and blood and function of the relevant organs. Second, the acupuncture points are located in the sacral and abdominal bladder region. Depending on the anatomical location of the nervous system, EA located at sacral acupoints (BL32, BL33) can stimulate sacral nerve roots, subrectal nerves and pubic nerves.^[33,34]^ The application of modern medicine to localize the S3 neural foramen to locate the BL33 has good feasibility,^[35]^ and the S3 nerve root is the nerve pathway with the most significant stimulation of the bladder’s forced urinary muscle, and needling at BL33 not only activates the excitatory fibers of the parasympathetic nerves but also facilitates the micturition reflex by stimulating the S3 nerve to inhibit the urethral sphincter, and thus may contribute to the emptying of the bladder.^[36]^ Stimulation of the parasympathetic nerve centers in the sacral region S2 to S4 by electroacupuncture at these 2 acupoints has a mechanism similar to sacral neuromodulation^[37]^; in terms of the intensity of sacral innervation of the detrusor muscle by the sacral nerves, S3 is the strongest, S4 is the second strongest, and S2 is weaker, and moreover, in the treatment of elevating intravesical pressure in patients with urinary retention, bilateral sacral neuromodulation achieves a more satisfactory therapeutic effect than unilateral S3 stimulation, and these neural structures are important for the control of urination and defecation. Third, at acupuncture points located in the abdomen. Long-needle needling of abdominal acupoints (CV3, CV2) stimulates the pelvic plexus nerves and bladder contraction, reduces residual urine volume and frequency of incontinence as well as increases the volume of diurnal and nocturnal urination.^[38–40]^ Long needles have the characteristics of deep penetration, strong needle sense, etc. From the point of view of the path of the needle, the abdomen at the selected acupoints at the site of abundant muscle tissue, nerves, blood vessels are distributed in the deep layers of the muscle, through the deep stimulation of long needles to the pelvic plexus nerves in the pelvic cavity, is conducive to the direct play of the nerve on the pelvic floor muscle groups to promote the regulation of the urinary drainage from the anatomical level of long needles in the treatment of urinary retention after the HZ provides the feasibility of long needles. Analyzed from the innervation point of view, one of the CV3 is innervated by the T12-L1 spinal ganglionic nerves, and the bladder is innervated by the T12-L2 and S2-S4, thus making the CV3 a commonly used and advantageous acupuncture point for the treatment of urinary obstruction in clinical practice. In addition, acupuncture at SP6 in the distal lower limb can increase the ATP content of the bladder and indirectly excite the pelvic nerves innervating the detrusor muscle.^[41]^ The acupuncture effect is also closely related to the overlapping effect of innervation under different acupoints,^[42]^ and the regulation of the bladder is mostly manifested as excitatory effects such as strengthening the contraction of the detrusor muscle and elevating the intravesical pressure of the bladder.

Clinical observations suggest that the duration of visceral dysfunction does not appear to be related to the severity of the rash, that AUR usually occurs at the beginning or during the development of the rash, and that urodynamic findings indicate the presence of diminished contraction of the detrusor muscle in the patient. By way of comparison, it has been found that attempts to remove catheters are more likely to fail in men because their urethra is longer than that of women thereby requiring a longer recovery cycle.^[43]^ Some scholars believe that the prognosis of acute urinary retention secondary to herpes zoster is usually self-limiting, and the prognosis of osteomyelitis-associated urinary and bowel dysfunction has been reported to be favorable, with the majority of patients fully recovering after a few weeks.^[8]^ But instead, clinically reported cases and the patients we encounter often presented with persistent neurogenic underactive bladder, with multiple attempts to remove the catheter ending in failure, and improving only after receiving treatments such as medications and acupuncture. Due to the insidious nature of the onset of the disease and the fact that antiviral treatment cannot completely prevent long-term sequelae, once the patient’s neurological symptoms such as dysuria and constipation do not improve in a timely manner after a short period of time after the onset of the disease, it is recommended that he undergoes urodynamics and receives acupuncture as soon as possible in order to capitalize on the proactive timing. The treatment focuses on the stimulation of the S3 sacral nerve to regulate the urinary nerve reflexes. Clinically, acute urinary retention symptoms can completely disappear after acupuncture treatment, which can be confirmed by the change of urodynamic parameters before and after the treatment, and WFmax as a value index for evaluating the bladder contractility improved significantly before and after the treatment in this patient. In addition, the fact that he maintained the ability to urinate spontaneously at the 12-month follow-up after the treatment showed that the effect of acupuncture did not diminish over time.

## 4. Conclusion and outlook

In this treatment study, we report an elderly patient with AUR secondary to VZV infection who fully recovered from acupuncture treatment after conventional drug therapy failed to work, and we appropriately prolonged the treatment cycle during the treatment period despite his recurrent condition, thereby reducing the likelihood of re-insertion of a catheter, and improving the outcome so that he had more benefit and improved his quality of life. To some extent, it can be considered that acupuncture may be a safe and promising complementary treatment option for ES, and early application of acupuncture should be considered clinically to avoid repeated catheters installation. However, this case has some limitations and lacks direct etiologic evidence. Therefore, confirmation of its effectiveness needs to be further evaluated through a large and multicenter prospective study to verify the clinical effect of acupuncture as a treatment for AUR triggered by herpes zoster. This article follows the CARE guidelines.

## Author contributions

**Data curation:** Zilong Tan.

**Investigation:** Zilong Tan.

**Methodology:** Jianwu Shen.

**Project administration:** Jianwu Shen.

**Writing – original draft:** Zilong Tan.

**Writing – review & editing:** Jianwu Shen.
